# Self-Directed Learning for Histopathology Using a Survey-Based Approach and Development of an Identification Key

**DOI:** 10.1007/s40670-025-02288-w

**Published:** 2025-01-24

**Authors:** Femke Simmer, Elisa Vink-Börger, Iris D. Nagtegaal

**Affiliations:** Department of Pathology, Radboud University Medical Center, P.O. Box 9101, 6500 HB Nijmegen, The Netherlands

**Keywords:** Histology, Education, Colorectal precursors, Identification key

## Abstract

For the classification of colorectal polyps, we implemented an approach in which students are encouraged to use their imagination. First, students received surveys with histological pictures via an online tool, had to create associations for the observed tissue structures, and use these in an identification key. Subsequently, an interactive lecture linked their descriptions to correct terminology, followed by an exam. Participation in the surveys varied between 69 and 100%. All students took the exam, with a 73% pass rate. Feedback from 62 students (84%) was positive, highlighting the method’s effectiveness, making our approach successful, and a blueprint for future educational innovations.

## Background

Over the years, the teaching of histopathology has changed from using glass slides and microscopes towards digital microscopy [1]. Nevertheless, learning histopathology is still perceived as difficult. Typically, students are directed to structures and are required to memorize the correct biomedical terminology. To independently recognize the histologic characteristics, students need several rounds of instructions. Unfortunately, there is an overall decrease in the number of contact hours for histology [2].

Teaching approaches that engage students will improve learning outcome and are superior to a teacher-centered approach. For example, interactive e-learnings and flipped classroom are used to increase student engagement. Importantly, this also stimulates critical thinking and problem solving in addition to reaching the main learning goals [3, 4].

According to the Cognitive Load Theory, it is important to tailor the amount of information in each learning activity [5]. This is, for example, addressed in pre-recorded video mini-lectures, in addition or instead of the traditional in-class lecture [6]. Such scaffolding approaches are effective for acquiring new knowledge and new skills. By breaking down a task into simple sequential steps, students are gradually guided towards becoming independent performers [7].

Visual literacy is the ability to interpret information from images. This skill is important in day-to-day life and even more for certain professions, including pathologists [8]. Encouraging students to find associations with images of familiar patterns and objects can facilitate processing of the new visual information. Dissection of the thinking process used for describing a simple object, photograph, or painting will also help [9].

We developed a colorectal polyp learning module to promote more engaged, independent, and productive learning, implementing these concepts. In brief, daily short surveys with histological images stimulated students to discover their own associations and lead to a personalized classification system. Here, we report the set-up, give examples of the students’ refreshing descriptive terms, show results and experiences, and give our future perspectives.

## Activity

### Setting

The learning module is part of “Screening as early diagnostics” in the second year of the bachelor of the Biomedical Sciences program of the Radboudumc in Nijmegen, The Netherlands. In this course, we use the Dutch population-based bowel cancer screening program as an example. This voluntary module focusses on the histology and classification of precursor lesions. The different relevant entities (conventional adenomas, traditional serrated adenomas, sessile serrated lesions, hyperplastic polyps) are linked to colorectal carcinogenesis. We use these examples to emphasize the role of pathology in clinical practice.

### Design

**Fig. 1:**
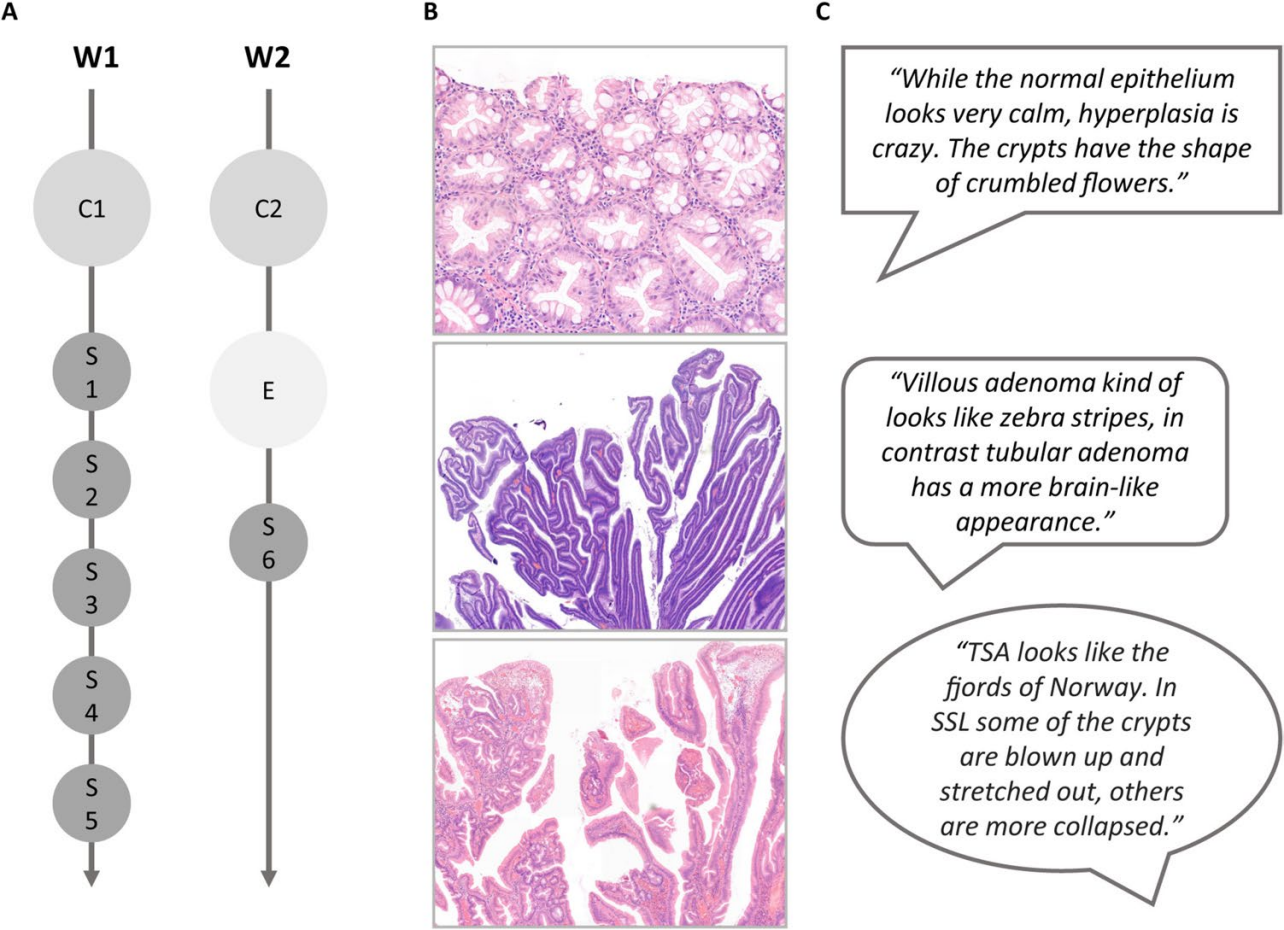
Overview of the learning module for classification of colorectal polyps. **A** Timeline with in-person contact moments (C), surveys (S), and exam (E) distributed over two weeks (W). C1 is the general introductory lecture; C2 the interactive lecture addressing the correct terminology; S1-5 contained histological images; S6 was to assess students’ satisfaction. **B** Histological images from the surveys. From top to bottom: hyperplastic polyp; villous adenoma; traditional serrated adenoma. **C** Examples of observations for the images in **B**

**Fig. 2:**
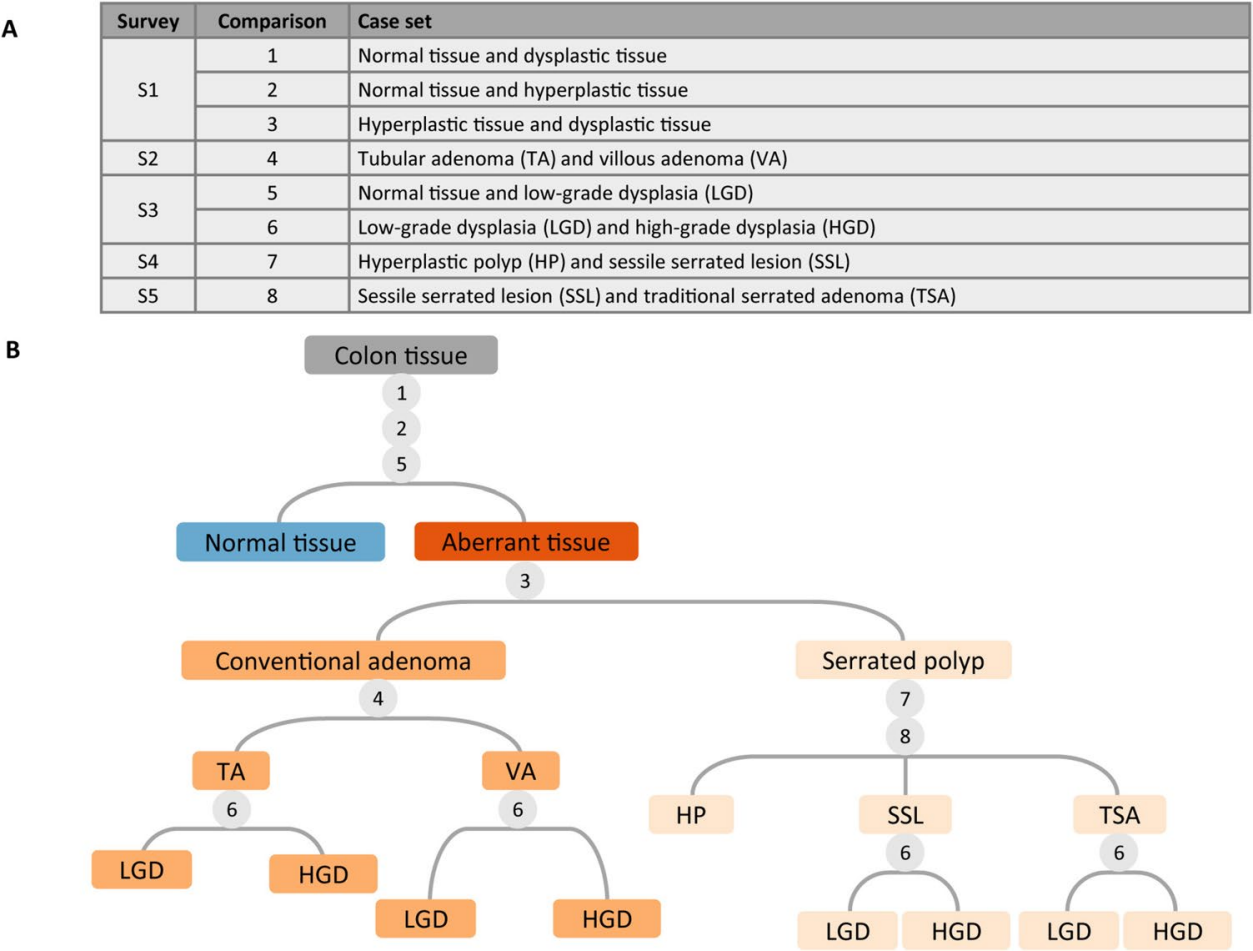
Generation of the identification key. **A** The five surveys with histological images. **B** The identification key for stepwise classification of colorectal precursor lesions. The numbers (1–8) correspond to the comparisons in **A**. At each number, the student should generate decisive questions about the histological features of the tissue

Figure 1A provides an overview of the new design. The activities are distributed over 1.5 weeks. After a general introductory lecture, describing the basis of colorectal cancer development and population screening, the procedure for learning the classification of polyps was explained to the students. Additionally, a description of the interactive way of learning histology was uploaded to the learning platform Brightspace (D2L). Directly after the lecture and the following days, the students received an email with a link to a survey using LimeSurvey (LimeSurvey GmbH). The surveys contained sets of static histological images that had to be compared. In Fig. 2B, the comparisons per survey are listed. The histological images were collected from the archives of the Pathology Department, Radboudumc, Nijmegen, The Netherlands, with consent for their use obtained through the opt-out method. The students were instructed to describe the differences between two entities in their own words, without focussing on proper terminology. They were asked to concentrate on the histology of the epithelium and to identify patterns. The surveys offer flexibility since they can be completed any time on the day they were received, or even at the end of the week, and should only take up maximally 15 min per survey. Using their descriptions from all surveys, the students had to create an identification key. Identification keys are typically used to identify biological entities like plants and animals and consist of a fixed sequence of identification steps. At each step, the user answers questions about one or more feature of the entity, which determines the next step. Students were provided with the basic identification key structure based on the WHO classification of colorectal precursor lesions and were asked to add questions and answers. After construction of the identification key, an interactive lecture addressed the correct terminology. The assessment was a short exam on the same platform as the surveys where the students classified new histological images. The exam score was the sum-of all correctly classified images (maximum 10; pass at 6 correct answers). Finally, student feedback was obtained with a survey of seven questions. For five questions, a 5-point Likert scale was used with 1 representing “strongly disagree” and 5 “strongly agree.” One question required a yes/no response, and another allowed free text. All responses to the assignments and questionnaire were collected anonymously.

## Results

The number of participants varied between 51 (69%) (5th survey) and 74 (100%) (exam). In the first survey, we compared normal colon tissue with dysplastic and hyperplastic polyps (Fig. 2A). Normal tissue was compared with flowers and hyperplastic changes with stars. Dysplasia was described as unorganized, stacked cells that differ in size and shape, and it was noted that the tissue looked more purple. The sessile serrated lesion in survey 5 looked like trees on top of a sphere. Figure 1B shows examples of the histological images used and Fig. 1C shows examples of the students’ descriptions.

Using the descriptions from each survey, the students had to formulate decisive questions about the histological features of the tissues and add these to the template of the identification key. The first node decides whether the tissue is normal or aberrant, followed by the distinction of the conventional and serrated pathway. Subsequently, the exact type of the polyp is determined, while in the fourth step, the grade of dysplasia is assessed (Fig. 2B).

Fifty-four students (73%) passed the exam, and three students (4%) obtained the maximum score. Almost all students recognized a sessile serrated lesion (93%). The hardest was a low-grade tubular adenoma, with 42% correct answers.

Students’ satisfaction was assessed with the last survey, which was completed by 62 students (84%). The method facilitated well in achieving the educational objective (38 students selected ≥ 4 of the rating scale). Forty-eight students would like to see this method in other courses in the future. Free text responses concerning improvements were categorized into themes: organization, instructions, feedback, and accompanying study materials. Seventeen students thought the instructions at the start were somewhat unclear, but that the goals and tasks became clearer during the course.

## Discussion

Here, we describe a new method to learn the classification of colorectal polyps using an identification key. By using a sequence of short surveys, we staggered the amount of available information. In this way, the students learn the structured process that is needed for a proper classification. Moreover, by giving a relatively small task each day, we aimed to reduce the cognitive load, keep the student motivated, stimulate students to repeatedly study the subject, and improve learning outcomes [10].

Our approach gives students control over their learning experience (self-directed). Encouraging students to use their own imagination and associations enhances motivation and engagement. Additionally, the approach stimulates critical thinking and problem solving, which are crucial skills for becoming a lifelong learner [11]. A potential drawback is the limited interaction with the teaching staff and peers. Our approach may not suit every student due to differences in prior knowledge, visual literacy, and preferred learning styles. However, this is true for every approach. We believe that a combination of different methods, including ours, should be integrated throughout the curriculum. Our approach requires limited resources, both in terms of hardware, software, and teaching staff, which is beneficial given the time constrains in the curriculum and high diagnostic workload of pathologists. Lastly, the use of static images is advantageous, because it focuses on specific areas, reducing cognitive overload compared to analyzing a whole digital slide.

In conclusion, we have developed a simple survey-based approach for classification of colorectal polyps. This approach provides engaged and independent learning, which was positively received by the students, and additionally has great potential for further innovations in the teaching of histopathology within our curriculum.

## Data Availability

The data generated and analyzed for this short communication are available from the corresponding author on reasonable request.
